# Round the Hospitals

**Published:** 1918-07-20

**Authors:** 


### ROUND THE HOSPITALS.

It was a truly historic occasion when, on July 15, the Queen with Princess Mary visited the headquarters of her Needlework Guild at Friary Court, St. James's Palace, to receive the Silver Wedding Shower of Gifts. The gifts, exclusive of sixty-six cases from overseas still on their way, numbered over half a million, and are spontaneous offerings collected without public appeal by branches of Queen Mary's Guild in every part of the globe. The amount of human interest packed into these offerings would fill many volumes if touched by a sympathetic pen. The very list of the countries contributing illustrates to what an extent the ideal home life of our Sovereign and his consort has stirred the world; how insensibly the British Royal Family has become a focus wherein is collected motions of compassion for the vast misery of this war to radiate forth in practical benefit to the suffering. The gifts were presented to Her Majesty by Princess Beatrice, and the Queen in reply expressed her delight that "the sailors and soldiers and airmen will benefit so largely from this charming and practical expression of good will to myself."

France's Day was a triumphant success everywhere. The Anglo-French Committee of the British Red Cross was united early this year with the old Committee of the French Red Cross in order to co-ordinate numerous agencies for the relief of the

**ROUND THE HOSPITALS—(*continued*).**

French wounded which have their headquarters in this country, and the tale of what is being accomplished testifies forcibly to the spirit of brotherhood which animates the societies. The Committee manage and maintain six hospitals, aggregating 1,400 beds, besides subsidising French hospitals in the poorer regions. Some 2,500 French hospitals have received gifts through this agency. The Prisoners of War Department sends 5,000 packages a month to French prisoners in Germany; other sections care for the tuberculous, the maimed, and the blind; substantial assistance is given to widows, orphans, and helpless refugees. The French Flag Nursing Corps has sent nearly 700 British nurses to French military hospitals; the British Committee has sixty canteens equipped behind the lines for the refreshment of hungry, thirsty, and tired *poilus*; it manages four of the British ambulance convoys working in the front lines, and has dispatched some 500 ambulances and cars from one end of France to the other, with voluntary English drivers—women or unfit men. With all this the need for more and more help grows as time goes on. No nation in the world has ever suffered more cruelly than France, and her sufferings increase hour by hour in this new and perhaps supreme assault.

THE authorities of the Nightingale Training-School at St. Thomas's Hospital have decided to admit as special probationers, without fees, V.A.D. nursing members and special military probationers who have served in military hospitals for a consecutive period of not less than two years and who desire to become trained nurses and to enter Q.A.I.M.N.S. This will be a great boon to some who have developed a decided vocation for nursing in the course of their military experiences. The position of special probationers in the Nightingale School is one of the most advantageous that can be held by women preparing for the higher posts in the nursing world. It is tinged by no "lady-like" dispensations from thoroughness in the preliminaries, but offers opportunity from the very beginning for close and concentrated study such as is too often impossible in otherwise well-organised training-schools. After a short preliminary training these probationers will be admitted to a course of three years' training, and, after passing the usual examinations, will be granted a full certificate.

THE London University, under the Ratan Tata Department of Social Science, is inaugurating a special course of training (October to July) for men and women who wish to devote themselves professionally to public administration or social work. Students will work under the direction of supervising tutors, and their training will comprise both theoretical and practical branches. The course is particularly well suited to fill the gap between leaving school and obtaining training as a nurse with the aim of taking work as health inspector. But, in order to meet the aims of such as come thus prepared to learn the art of nursing, the nurse-training schools should be able to offer facilities for keeping up the knowledge already acquired and for deepening it in special directions. The need for co-ordinating hospital training with social service and administrative courses of study is urgent and widely felt.

SOME of the benefits conferred on London by the Council nurses are strikingly illustrated by Sir Robert Blair in his reply on the report from the medical officers of the Board of Education on the London County Council's school medical services. The efforts to improve the cleanliness of the school children have proceeded with unabated vigour during the war. The number of children who have received treatment at the centres has more than doubled in this period, and is now over thirty thousand, and after-treatment is carried on under an admirable system of co-ordination, whereby necessitous children are selected for special nutrition. We look forward to the time when the laborious and more than commonly trying duty of the school nurses in ensuring bodily cleanliness among the children will be crowned with complete success, and the louse become as extinct as the dodo.

AN interesting election took place in the Manchester Centre of the College of Nursing on the retirement of Miss Smith, R.R.C., from the position of local representative. Six ladies were nominated to succeed her, viz.: Miss Burgess, R.R.C., Crumpsall Infirmary; Mrs. Burgess, M.B.E., Victoria Park, Manchester; Miss Fletcher, R.R.C., 2nd Western General Hospital; Mrs. Caddum, late matron, Ancoats Hospital; Miss Pilgrim, County Inspector, Queen's Nurses; Miss Salisbury, 2nd Western General Hospital, Levenshulme. Of these Miss Fletcher received the highest vote, and she was accordingly appointed local representative. The local hon. secretary is Miss Maud Earl, matron, Ancoats Hospital, Manchester, and she requests all members of the College residing within the East Lancashire area to communicate with her. The fee for joining the Centre has not yet been agreed upon. It will be discussed after the next meeting of the Centre, on July 23.

THE Cornwall Centre of the College of Nursing held a good meeting on July 11 at the Royal Cornwall Infirmary, Truro. A large and keenly appreciative audience assembled to hear Mr. Panting, F.R.C.S., on "The Treatment of War Wounds and Conditions." At the conclusion of his address Miss Kimmerly, County Health Visitor, gave an interesting account of the work done in the county by midwives and health visitors. Tea was served after the meeting, and it was unanimously agreed that the new Centre was already proving a professional stimulus and source of social cheer to Cornish nurses. The next meeting will be held at Bodmin early in October. Meantime, members must work hard to draw in all nurses who are qualified to become members of the College of Nursing.

**ROUND THE HOSPITALS**—(*continued*).

It is not surprising that applications for admission to the Edith Cavell Homes of Rest for Nurses are coming in fast just now. Coombe Head, near Haslemere, the first fine property acquired for the purposes of the Trust, accommodates 100 nurses a year on the estimate of a month each, and Little Wych, near Bridport, will take eighty. We understand that new homes are being opened to meet the existing need, for there has probably never before been so large a number of tired nurses. Applications should be sent to the Hon. Secretary, 25 Victoria Street, S.W. 1, where much-needed subscriptions will be welcomed.

A WRITER in the *Spectator* quotes from "Florence Nightingale to her Nurses," a little volume published in 1915, the well-known passage in which she describes the generous ministrations of German women to the frozen and starving French prisoners, who, to the lasting disgrace, it may be said, of the military authorities, were carried to Germany during the hard winter 1870-71 in open cattle-trucks, exposed without food or covering for many days. It is one of the abominable methods adopted by German war propagandists, in order to crush pity in their men and women alike, that for many years past they have striven to hold up to scorn as unpatriotic and "sentimental" (much-abused word) the natural movements of pity shown by German nurses and other women for the wounded who fell into their hands in that war. Efforts were persistently made to represent in the most contemptible light the noble compassion of the nurses who extended their care to wounded enemies, and Germans have been accustomed to refer to it in conversation as degrading. We can now, in the behaviour of the new generation of German women, behold the result of calling good evil and evil good.

THE comparative tables which the British Hospitals Association collected showing the amount of soap and alkali used in washing each 1,000 articles ought to be widely studied by matrons of hospitals which possess private laundries. They open up a profitable source of investigation. One laundry, for instance, used 2.5 lb. of soap, others 11 lb., 12 lb., 19 lb. for the washing of every 1,000 pieces. As regards alkali, the amounts varied from 3.5 lb. (where a water softener was in use) to 8.4 lb., 11 lb., 13.5 lb., 20.3 lb. In all these cases the water is reported "soft." In monetary value the cost for soap of each 1,000 articles washed varied from 2s. 10d. to 26s. In one workhouse infirmary converted into a military hospital 415,000 articles were washed in six months at a cost for soaps and alkali of over £200. "If the washing had been done on a controlled and scientific basis their bill should not have exceeded £30." Problem for all laundry managers to solve: "How much soap and alkali do you use to wash every 1,000 articles? Could this amount be reduced? How much could be saved by reducing it?"

THE hard case of the Army masseuse is pleaded in the *Times* by "The Mother of a Masseuse." The pay is £2 10s. a week, with uniform allowance of £4 a year; no board or lodging. Masseuses in the Navy get £3 a week, and on the face of it there is good reason for levelling up the pay of Army masseuses to the same sum. A petition to this effect which was sent up to the War Office early in the year is still under consideration, and it is more than time that the matter was settled.

WE note in the recent report of the Middlesex Hospital for 1917 the retirement on her pension of Miss Langridge, sister of Queen Mary Ward, after over twenty years' service, fifteen of which were spent in the Cancer Charity. The report states: "The whole of her career at the hospital was marked by intense devotion to the patients under her care, and all her actions were influenced by a sincerity of purpose and a true spirit of helpfulness which brought bodily comfort and ease of mind to the many who turned to her in their hour of trial. She was an ideal sister in the fullest sense of the word."

SISTER AMY MARION PARRY, of Poulton-le-Fylde, has been exposed to serious annoyance in consequence of accepting the post of "nursing officer and lecturer" in the local St. John Ambulance Brigade. Some officious, and probably weak-minded, individual wrote an abusive letter and post-card to Dr. Buckley, the divisional surgeon, introducing Sister Parry's name, and the doctor, hastily and without evidence, concluded that she was the culprit. No inquiry was held into the circumstances, nor was Miss Parry afforded an opportunity of denying the authorship of the writings complained of. She was suspended from her office by the Deputy Commissioner of the St. John Ambulance Brigade, and brought an action in the Blackpool County Court claiming £10 10s. damages and applying for an injunction against Dr. Buckley. The Judge gave judgment for the defendant on the technical point that the defendant, in suspending Sister Parry, had acted on the instructions of the Deputy Commissioner, but he expressed his cordial sympathy with the plaintiff. We are glad to say that the defendant withdrew every imputation made against Miss Parry, offered to reinstate her, and refused to ask for costs. Nevertheless, it has been a severe ordeal for her. Every effort should be made to discover the real author of the anonymous letters with which Poulton-le-Fylde is said to be deluged.

THE Inquiry Depôt for the Wounded and Missing, 18 Carlton House Terrace, S.W. 1., is appealing for voluntary aid in clerical work during the summer months, in order to afford their regular staff a much-needed holiday. It is work which, from the nature of things, can never slacken. It is not difficult to master, and part-timers will be welcomed.

**ROUND THE HOSPITALS—(*continued*).**

HANDSOME additions have lately been made to the nurses' salaries by the committee of the Taunton and Somerset Hospital. Probationers' salaries, which were formerly at the rate of £5, £10, £15, and £25, have been raised to £12, £18, £24, and £30. Nurses remaining after their training is completed, and joining the private nursing staff, now receive £40 from the beginning.

THE Waste Tinfoil Collection for hospitals, inaugurated in the first year of the war by the Ancient Order of Druids, has now issued its report, and a very satisfactory document it is. Through the collection of tinfoil from packets of chocolates, cigarettes, etc., close upon 100,000 lb. of this precious commodity, so valuable for munition purposes, has been rescued from the dustbin. The monetary value of the stuff when sold amounted to £1,363, with which four beds have been endowed in various hospitals, the latest being one in the Royal Sussex County Hospital at Brighton. Plenty of tinfoil runs to waste in all institutions. Any who will help in this anti-waste campaign, designed in the coming year to endow yet another hospital bed, should start at once and send their tinfoil, as soon as it becomes a bulky parcel, to the Central Collecting Depôt, 1 Little Crown Court, Wardour Street, London, W. 1.

THE Worcester Guardians had before them recently a statement from the Inspector of the Local Government Board to the effect that the superintendent nurse of the infirmary was now dispensing the medicines, and asking if she were qualified to do so. Dr. Fowler, who had inspected the nursing wards at the request of the Inspector, expressed the opinion that since the nursing staff had been reduced the nurses had been overworked, especially the superintendent nurse, who did not get sufficient leisure to maintain her health. The superintendent nurse, who was called in, stated she did the bulk of the ordinary dispensing under the direction of the doctor and to help him, but was not qualified to dispense. The matter was adjourned for further inquiry. No one appears to have suggested, however, that the superintendent nurse was entitled to extra pay for the heavy extra duties imposed on her.

THE nursing staff of the Royal Prince Alfred Hospital, Sydney, having been invited to confer with the medical superintendent, the secretary, and the matron on some outstanding points of difficulty, certain recommendations were made to the board which brought about several improvements in the conditions of the nursing service. The first was the raising of the salaries of fully qualified nurses who are retained on the staff to the following rates: sisters, for first year, £100; for second year, £110; for third year, £120; and charge nurses, £80. The latter class have a chance of being appointed sisters after one year's service. The course of nurses' training remains at four years, but nurses wishing to proceed on military service may obtain a certificate of three and a half years training on condition that, if the opportunity shall be available, they shall return to complete their full course. For the future all applicants for training as nurses, who shall be selected as suitable, shall be given a month's trial at the hospital. If they are considered satisfactory they will return to their homes and will enter for training on May 1 or November 1, as may be decided. By this means all nurses will come on to the staff in batches and will receive their lectures and complete their training together. The new regulations contain also liberal arrangements as to sick-pay, leave, and the like, and the whole scheme is in keeping with the reputation for enlightened management that belongs to this great Australian hospital.

ALL institutions which keep pigs ought to look around for bracken in their neighbourhood. While the fronds above ground are found to be neither palatable nor wholesome, the rhizomes or creeping stems below the surface have been proved by Professor James Hendrick to yield a rich store of nutriment highly approved by all pigs who have not been rendered dainty by a diet of rich concentrated foods. If the pigs are turned out unringed on bracken land they will root it out for themselves.

THREE thousand women have been recruited from schools and colleges by the Women's National Land Service Corps to harvest the flax, and are being sent down to camp in Somerset for preliminary training. They will be under semi-military discipline and are to sleep seven in a tent. Each camp has a hospital tent attached, with nurses who have volunteered from various hospitals to spend their leave in this manner.

A NEW leaflet on the preparation of buttermilk cheese, No. 324, has now been issued by the Board of Agriculture, and may be obtained free on application to the Board at 3 St. James's Square, S.W. 1. From buttermilk alone a smooth and creamy cheese can be prepared, which will keep about a week. Another kind, which keeps longer, can be made from two-thirds buttermilk and one-third new milk, and a pressed cheese from separated milk and 20 per cent. of buttermilk. By making these cottage cheeses the waste of milk involved in butter-making can be wholly obviated, and welcome variety introduced into the institution.

